# Identification of novel membrane markers in circulating tumor cells of mesenchymal state in breast cancer

**DOI:** 10.1016/j.bbrep.2024.101652

**Published:** 2024-02-13

**Authors:** Yongdeuk Hwang, Yurim Kim, Jiin Min, Jinmyung Jung

**Affiliations:** Division of Data Science, College of Information and Communication Technology, The University of Suwon, Hwaseong, 18323, Republic of Korea

**Keywords:** Circulating tumor cells, Mesenchymal state, Membrane marker, Breast cancer

## Abstract

Cancer metastasis is a major cause of cancer-related deaths worldwide. The ability to detect and monitor circulating tumor cells (CTCs) offers a promising approach to early detection and management of metastasis. Although studies on epithelial markers for CTC detection are actively underway, the discovery of mesenchymal markers has not been studied sufficiently. In this study, we developed a new pipeline to identify membrane markers in CTCs of mesenchymal state in breast cancer based on expression profiles of the 310 CTC samples. From the total CTC samples, only CTC samples in the mesenchymal state were collected by employing hierarchical clustering. In samples belonging to the mesenchymal state, we calculated the correlation coefficients between 1995 membrane genes and ZEB2, which was determined as the key mesenchymal signature, allowing the 84 positively correlated genes. Furthermore, to ensure clinical significance, Kaplan-Meier analysis were performed on the 124 breast cancer patients, resulting in the 14 genes predicting prognosis. By exploring genes commonly identified in the both analyses, F11R and PTGIR were characterized as membrane markers in CTCs of mesenchymal state in breast cancer, which were evaluated by enriched terms, literature evidence, and relevant molecular pathways. We expect that the results will be helpful to more effective strategies for metastasis management.

## Highlights

• We developed a new pipeline to identify circulating tumor cell (CTC) markers of mesenchymal state in breast cancer.
• A subgroup containing only CTCs in mesenchymal state was obtained by using hierarchical clustering.
• We explored genes that correlated with ZEB2 and showed clinical significance, characterizing F11R and PTGIR as new markers.

## 1 Introduction

Circulating tumor cells (CTCs) are a type of cancer cells that have detached from the primary tumor and have entered the vascular system [1]. These cells are of particular interest in the field of oncology due to their potential role in cancer metastasis [2]. The detection of CTCs offers several advantages over traditional tissue biopsy methods. One of the most significant benefits is its non-invasive nature, which allows for repeated testing without causing discomfort or harm to the patient. Additionally, CTC detection provides comprehensive information about the risk of cancer metastasis and progression, making it a valuable tool in cancer management [2,3].

CTCs detection technology has been applied to various clinical applications, including cancer diagnosis, prognosis, and therapeutic response monitoring. CTCs are used in many cancers as a surrogate biomarker to predict clinical benefit. Studies have been conducted on various cancer types, including breast cancer. In Jin et al.‘s study, CTCs were detected in 50%–81% of patients with early stage breast cancer [4]. Magbanua et al.‘s study showed a positive correlation between CTC detection and reduced distant recurrence-free survival (DRFS) [5]. Wang et al.‘s study found that fewer CTC counts were detected in the surgical group than in the trastuzumab group [6]. The CTC applications mentioned above utilize epithelial makers such as EpCAM and cytokeratin.

A widely accepted method for detecting CTCs involves the use of membrane markers that are highly expressed in these cells. Epithelial Cell Adhesion Molecule (EpCAM) is one such membrane marker that is commonly used for this purpose. EpCAM has been extensively utilized to detect CTCs in cancers of epithelial origin, including but not limited to breast cancer [7]. However, a challenge arises when CTCs undergo a process known as epithelial-mesenchymal transition (EMT). During EMT, epithelial markers such as EpCAM are down-regulated, which subsequently reduces the efficiency of detecting CTCs using these markers [8]. To address this issue, researchers have considered using not only epithelial markers but also mesenchymal markers [9]. Several mesenchymal markers have been identified, which are highly expressed during EMT process, such as vimentin, *N*-cadherin, fibroblast-specific protein1, ZEB, and fibronectin [9]. However, most of them are located in the cytoplasm or nucleus rather than on the cell membrane, making it difficult to use them as detection markers for CTCs in the mesenchymal state [8].

In light of these challenges, our study aimed to identify novel membrane markers in CTCs of mesenchymal state in breast cancer. The reason why breast cancer was chosen as the target cancer is because its high prevalence continues to cause significant suffering to numerous women [10,11]. Although extensive efforts have been made to detect breast cancer at an early stage prior to metastasis, it is often diagnosed post-metastasis [12]. Enhancing the accuracy of CTC detection in patients with breast cancer could potentially facilitate earlier diagnosis and improve prognostic outcomes.

**Fig. 1:**
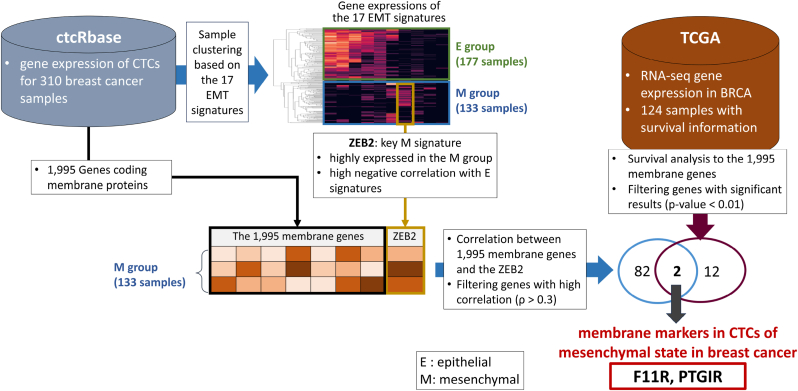
Strategy overview. From ctcRbase, expression profiles of 1995 membrane genes in 133 breast CTC samples of mesenchymal state were analyzed by computing correlations with ZEB2 (i.e., a key mesenchymal signature). This analysis yielded the 84 positively correlated membrane genes. From TCGA, expression profiles of 1995 membrane genes in BRCA samples were explored using survival analysis to ensure clinical significance, which produced the 14 significant membrane genes. Finally, F11R and PTGIR that are commonly identified in both analyses were characterized as novel membrane markers in CTCs of mesenchymal state in breast cancer.

In this study, RNA-seq expression data of breast CTC samples were mainly used, which were clustered into two groups based on the known 17 EMT gene signatures of breast cancer. The clustering process was performed to collect only CTC samples in the mesenchymal state from the total CTC samples that include the both epithelial and mesenchymal states due to the EMT process. This clustering process was necessary because our goal was to identify membrane markers that facilitate to increase the detection efficiency of CTCs in the mesenchymal state. In samples belonging to the mesenchymal state, we identified membrane markers that were not only highly correlated with the ZEB2 but also showing clinical significance. As a result, we characterized F11R and PTGIR as novel membrane markers in CTCs of mesenchymal state in breast cancer (Fig. 1).

## 2 Materials and methods

### 2.1 Data preparation and preprocessing

CtcRbase is a database that contains gene expression data of circulating tumor cells (CTCs) for several kinds of cancer types, such as breast, colon, liver, and so on [13]. From the database, 310 CTC samples of breast cancer were collected from six RNA-seq expression profiles, i.e., GSE86978 (77 samples), GSE111065 (69 samples), GSE109761 (59 samples), GSE75367 (61 samples), GSE51827 (29 samples), and GSE67939 (15 samples). The 310 CTC samples were merged and preprocessed using the following three techniques sequentially: 1) gene expression averaging per gene by mapping to gene symbols, 2) a logarithmic transformation on all expression values to minimize outlier effects, and 3) Z-transform and quantile normalization to allow equal expression distribution for each sample.

The 17 epithelial to mesenchymal transition (EMT) gene signatures in breast cancer were collected from a previous study performed by Alsullman's group [14], which were used to cluster the 310 CTC samples into two subgroups and determine their characteristics (i.e., epithelial or mesenchymal state). By literature curation, the 17 signatures were classified into two subgroups, such as ten mesenchymal signatures (FN1, FOXC2, MMP2, MMP3, SNAI1, SNAI2, TWIST, VIM, ZEB1, and ZEB2) and seven epithelial signatures (CDH1, CLDN3, CLDN4, CLDN7, DSP, SOX10, and TWIST2).

The purpose of this study is to increase the probability of detecting CTCs in the mesenchymal state, and membrane proteins are generally used in the detection process. Therefore, the 1995 genes coding cellular membrane proteins were collected from *membrane* term of cellular component in gene ontology database (GO:0016,020) [15].

### 2.2 Hierarchical clustering

We performed hierarchical clustering on the total of 310 CTC samples using the collected 17 EMT signatures to filter in only CTC samples in the mesenchymal state. The Euclidean distance was used for the distance between samples, and the average linkage clustering method was used for the distance between clusters. This process finally generated two subgroups (i.e., epithelial group and mesenchymal group). Hierarchical clustering analysis was performed by modules in *Seaborn* package [16].

### 2.3 Correlation analysis

The Pearson correlation coefficient is computed as below:

$$r=\frac{\sum \left(x-m_{x}\right)\left(y-m_{y}\right)}{\sqrt{\sum {\left(x-m_{x}\right)}^{2}{\left(y-m_{y}\right)}^{2}}}$$,

where $m_{x}$ is the mean of the vector x, and $m_{y}$ is the mean of the vector y [17]. In this study, vector x represents the expression level of ZEB2 (the determined key mesenchymal signature), and vector y is taken as the expression level of one of the 1995 membrane genes.

### 2.4 Survival analysis

Survival analysis was performed on breast cancer patients to identify markers presenting clinical significance. For this purpose, among the 891 BRCA patients in TCGA, we selected the 124 patients whose survival information of ‘*days_to_death’* is available. For each of the 1995 membrane genes, the Kaplan-Meier analysis were performed on the two subgroups (i.e., high and low expression) of the 124 patients, which were divided based on the median expression value of the corresponding gene.

## 3 Results

**Fig. 2:**
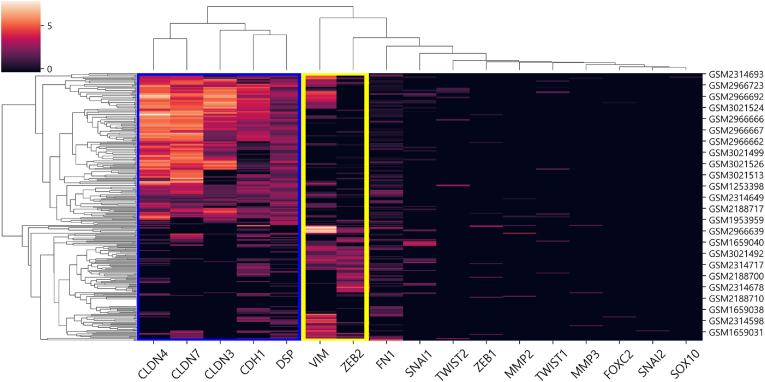
Results of hierarchical clustering to 310 samples based on the 17 EMT gene signatures. It generated two subgroups: the mesenchymal group with 133 samples and the epithelial group with 177 samples. We noticed that the two subgroups were clearly clustered with the seven signatures, i.e., five epithelial signatures represented by blue box and two mesenchymal signatures represented by yellow box. Out of the two mesenchymal signatures, ZEB2 was selected as the key mesenchymal signature for further analysis, as it produced higher negative correlations with the five epithelial signatures. (For interpretation of the references to colour in this figure legend, the reader is referred to the Web version of this article.)

Two subgroups were generated by performing hierarchical clustering on the gene expression profiles of the 310 CTC samples with the 17 EMT signatures (Fig. 2). We noticed that the two subgroups were clearly clustered with the seven signatures, which include two mesenchymal signatures (VIM and ZEB2) and five epithelial signatures (CLDN4, CLDN7, CLDN3, CDH1, and DSP). Here, the subgroup containing 133 samples was determined as the mesenchymal group, where two mesenchymal signatures were highly expressed and five epithelial signatures were less expressed, and the other subgroup containing 177 samples was determined as epithelial group. In further analysis, only the mesenchymal group was used, as the purpose of this study is characterizing membrane markers of CTCs in the mesenchymal state. Out of the two mesenchymal signatures (VIM and ZEB2), ZEB2 was selected as the key mesenchymal signature because it showed a higher negative correlation with the five epithelial signatures (CLDN4, CLDN7, CLDN3, CDH1, and DSP) (ρ = −0.46). Thus, we computed correlation coefficients between ZEB2 and the 1995 membrane genes. It resulted in 84 positively correlated membrane genes (ρ > 0.3), which are candidates of membrane markers in CTCs of mesenchymal state in breast cancer **(**Table S1**).**

**Fig. 3:**
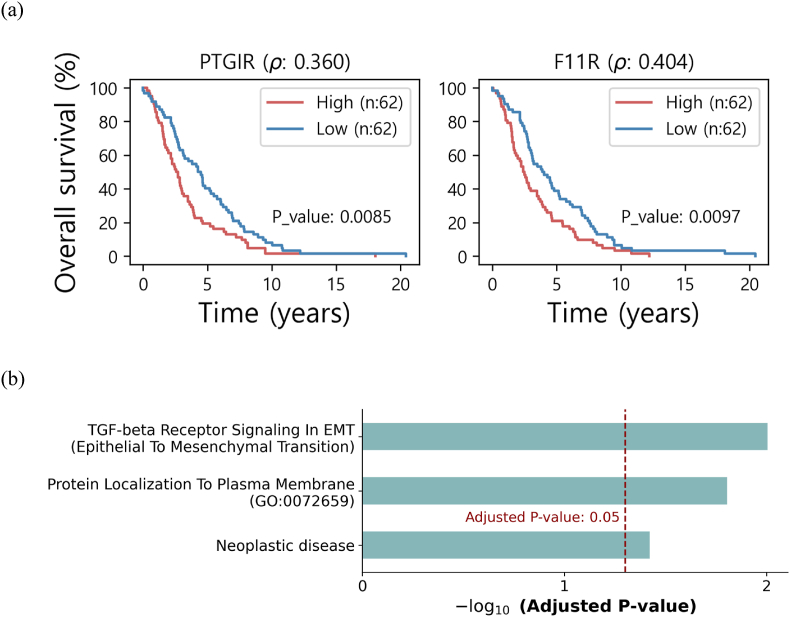
(a) Kaplan-Meier plots of the two mesenchymal membrane markers. ρ in subtitle indicates the correlation coefficient with ZEB2 signature (b) Enriched biological terms of the two mesenchymal membrane markers (PTGIR and F11R).

In order to secure a marker with clinical significance, the Kaplan-Meier analysis and the log-rank tests were performed on the 124 BRCA patients in TCGA for each of the 1995 membrane genes. As a result, the 14 membrane genes showed significant results (*P*-value <0.01 as well as showing worse prognosis in highly expressed samples) **(**Table S2**).** Finally, we explored genes that were commonly identified in the both analyses (correlation and survival analysis), characterizing the F11R and PTGIR as the membrane markers in CTCs of mesenchymal state in breast cancer. We noticed that the two characterized genes are receptors. In Fig. 3a, their survival graphs were depicted along with the correlation coefficient (ρ).

Two enriched terms associated with cancer metastasis were identified for the two characterized markers, which are *TGF-beta Receptor Signaling In EMT (Epithelial To Mesenchymal Transition)* in Reactome [18] and *Neoplastic disease* in DisGeNet [19] (Fig. 3b). As we expected, we found that *Protein Localization To Plasma Membrane* term was also among the enriched terms.

## 4 Discussion

Circulating tumor cells (CTCs) play a pivotal role in the metastasis of cancer, acting as a bridge between the primary tumor and distant metastatic sites. This critical role has led to an increased focus on CTCs in recent years, with numerous studies being conducted to enhance the detection performance of these cells. The importance of these studies cannot be overstated, as improved detection methods could lead to earlier diagnosis and more effective treatment strategies for cancer patients.

In this study, we developed a new pipeline to identify membrane markers of breast CTCs in the mesenchymal state. This pipeline involves in three strategies: 1) hierarchical clustering to divide CTC samples into two distinct subgroups (epithelial and mesenchymal groups), 2) correlation analysis with ZEB2 (a key mesenchymal signature) to identify candidates of novel membrane markers in CTCs of mesenchymal state, and 3) survival analysis to ensure clinical significance. As a result, we proposed F11R and PTGIR as new membrane markers for detecting CTCs of mesenchymal state in breast cancer.

Previous studies have shown that the characterized two markers (F11R and PTGIR) are associated with cancer progression, epithelial-mesenchymal transition (EMT), or metastasis. For instance, one study showed that F11R can induce EMT and serve as a prognostic factor for breast cancer [20]. F11R may be involved in cell migration by regulating epithelial adhesion junction signaling and EMT [20]. Another study showed that F11R is participated in various biological processes, including paracellular permeability, tight junction formation and maintenance, leukocyte *trans*-endothelial migration, epithelial-to-mesenchymal transition, angiogenesis, reovirus binding, and platelet activation [21]. Similarly, PTGIR activation was shown to be associated with inducing the expression of metastasis-related or pro-angiogenic genes [22].

**Fig. 4:**
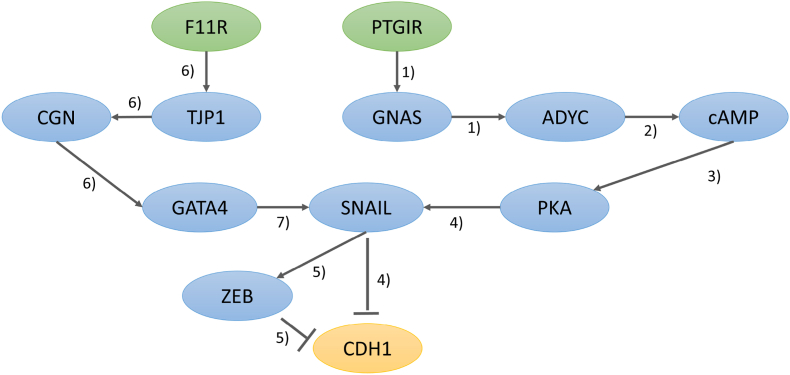
Molecular pathways from the characterized two markers (F11R and PTGIR) to CDH1. CDH1 (*E*-cadherin) is an essential transmembrane protein within adherens junctions, whose downregulation was found to increase the EMT process. The ZEB protein determined as a key mesenchymal signature in this study is also involved in this pathway. Reference: 1) [24], 2) [25], 3) [26], 4) [27], 5) [28], 6) KEGG pathway (Tight junction: map04530), 7) [23].

By exploring literature, we constructed molecular pathways from the characterized two markers (F11R and PTGIR) to CDH1 (*E*-cadherin). CDH1 is one of the most studied genes involved in the EMT process, whose downregulation was found to increase the EMT process [23] (Fig. 4). The pathway starting at PTGIR goes through GNAS, ADYC, cAMP, PKA, SNAIL and reaches CDH1 [[24], [25], [26], [27]]. The other pathway starting at F11R goes through TJP1, CGN, GATA4, SNAIL and reaches CDH1 ([23] and KEGG pathway: map04530). We noticed that the two pathways meet at SNAIL, which was found to suppress expression of CDH1 and induce detachment of primary mesenchymal cells through EMT process [23]. The ZEB protein determined as a key mesenchymal signature in this study is also involved in this pathway.

These findings provide valuable insights into the role of these genes in cancer progression and metastasis. Further research will be needed to fully understand their mechanisms of action and potential applications in cancer diagnosis and treatment. Most of all, it would be great if the results were experimentally proven; however, this was not possible in the given circumstances. By continuing to explore these and other potential CTC detection markers, we hope to contribute to the development of more effective strategies for metastasis management.

## Funding

This work was supported by a 10.13039/501100003725National Research Foundation of Korea (NRF) grant funded by the 10.13039/501100014188Korean government (MSIT) (NRF-2022R1C1C1008823).

## CRediT authorship contribution statement

**Yongdeuk Hwang:** Writing – review & editing, Writing – original draft, Validation, Methodology, Investigation, Conceptualization. **Yurim Kim:** Visualization, Software, Data curation. **Jiin Min:** Software, Resources, Data curation. **Jinmyung Jung:** W.

## Declaration of competing interest

The authors declare that they have no known competing financial interests or personal relationships that might affect the research reported in this paper.

## Data Availability

Data will be made available on request.
